# Clinical learning environment of nursing and midwifery students in Ghana

**DOI:** 10.1186/s12912-020-00533-8

**Published:** 2021-01-07

**Authors:** Florence Assibi Ziba, Vida Nyagre Yakong, Zakari Ali

**Affiliations:** 1 Department of Nursing, University for Development Studies, Tamale, Ghana; 2 Department of Midwifery, University for Development Studies, Tamale, Ghana; 3 Nutrition Theme, MRC Unit The Gambia at the London School of Hygiene and Tropical Medicine, Atlantic Boulevard, Fajara, P. O. Box 273, Banjul, The Gambia; 4 Department of Nutritional Sciences, School of Allied Health Sciences, University for Development Studies, P.O Box 1883, Tamale, Ghana

**Keywords:** CLES+T, Clinical placement, Learning environment, Nursing and midwifery, Ghana

## Abstract

**Background:**

Data on student experience of the clinical learning environment in Ghana are scarce. We therefore aimed to assess students’ evaluation of the clinical learning environment and the factors that influence their learning experience.

**Methods:**

This was a cross-sectional survey of 225 undergraduate nursing and midwifery students. We used the Clinical Learning Environment and Supervision + Nurse Teacher (CLES +T) evaluation scale to assess students’ experience of their clinical placement. The association between student demographic characteristics and clinical placement experience was determined using t-test or ANOVA.

**Results:**

Most of the sampled students were Nurses (67%) and in the third year of training (81%). More students received supervision from a nurse (57%) during clinical placement and team supervision (67%) was the most common during clinical placement. Nursing students were more likely to rate their clinical experience better than midwifery students (*p*=0.002). Students who had increased contact with private supervisors were also more likely to rate their experience higher (*p=*0.002). Clinical experience was also rated higher by students who received successful supervision compared to those who had unsuccessful or team supervision (*p*=0.001).

**Conclusion:**

Team supervision is high in health facilities where students undertake clinical placement in Ghana. Frequent contact with private supervisor and successful supervision are associated with better rating of clinical experience among Ghanaian undergraduate nursing and midwifery students.

**Supplementary Information:**

The online version contains supplementary material available at 10.1186/s12912-020-00533-8.

## Background

Nursing care is pivotal in the health care services worldwide. Globally, nurses and midwives constitute 59% of the health workforce [1]. In Ghana, out of the 115, 650 health workers employed by the government, nurses and midwives account for 58% [2]. The axillary nurses (community and enrolled nurses; trained for 2 years) make up the majority (53%) of the total nursing and midwifery workforce whiles professional nurses (with at least 3 years of training resulting in the acquisition of diploma, undergraduate, postgraduate qualifications, or specific speciality areas of study) account for 47% [2, 3]. The nurse serves as the main vessel that convey most interventions and care necessary for individuals utilizing health care services. For nurses to efficiently perform the myriads of duties, it depends on their ability to apply theory to practice [4, 5]. Hence, nursing training involves both theory and practical training. Each aspect of the training carries important weight.

The clinical skills acquisition of nurses is so important that the Nursing and Midwifery Council (NMC) of Ghana, a body mandated to regulate the training of nurses increased the clinical contact hours of students to 432 h, 624 h and 576 h for the first, second and third year of training respectively [6]. According to Benner [7], skills acquisition is not an event but a process where individual nurses start as novice and gradually become experts. Whiles the acquisition of knowledge on the theory is done in the classroom, the skills acquisition is done in the skills laboratory and clinical learning environment or setting depending on one’s level of study.

The clinical learning environment (CLE) is a complex and constantly changing setting [8]. The CLE can be influenced by several factors such as the kind of supervisor, the quality of feedback received by students, the context and the students [9, 10]. Much of skills teaching are done by the nurses in clinical practice regardless of their level of education and expertise.

This implies that the quality of clinical teaching of students depends on the efficiency of the supervisor they meet in the clinical facility [11, 12]. In Ghana, it is regarded the responsibility of every registered nurse or midwife to provide teaching or guidance for students who work with them on their shift without any specific training for that purpose. The registered nurses supervise students and do not receive payments by any of the institutions for this service. Some of these supervisors may not be adequately prepared or motivated for the task of clinical teaching [9]. According to Chan and Ip [13], their relationship is very key and determines the kind of learning environment created. A positive learning clinical environment is a result of good relationship between the supervisor and the students. Nursing students will always be motivated to learn in environments where there is mutual respect and students are involved in the team and supported with their decision making [4].

Constructs of the clinical learning environment positively influence students’ satisfaction with their learning activities [14]. The pedagogical atmosphere, ward manager leadership style and supervisory relationship are important factors that contribute to satisfaction with the clinical environment. Students who have the chance to meet a supervisor on more regular basis tend to well appreciate the contribution of the clinical environment to their skills learning. Having access to a supervisor or mentor allows the student to learn more and improve the skills learning.

Despite the importance of clinical learning for nursing students, it comes with some challenges to students, faculty and supervisors. For students, depending on the level of study it can be demanding especially when students feel, they lack the right skills for a particular assigned task [15]. They worry about the probability of supervisors holding negative perceptions about them which could affect their grading and therefore may come under “pressure” to please their supervisors [9].

Crowding of students in the clinical setting is a major challenge to clinical teaching and learning [16, 17]. Overcrowding in the ward makes it difficult for both students and clinical supervisors to engage meaningfully. When student numbers are high it means student-preceptor ratios will be higher than required for effective clinical learning. When students are more than the physical space can accommodate it becomes very difficult for staff to even assist them to learn clinical skills [18]. In Ghana, the ration of a supervisor to student is approximately 1: 10 or more students in a shift [19]. This is because students are required to do their clinical placement only in the hospital setting. Therefore, the need to improve clinical nursing education is an important aspect of training of nurses. Thematic areas of importance in improving clinical nursing education include having positive clinical environment, effective clinical supervision, adequate assessment of students and clinical-academic collaborations [20]. However, there are no current studies reporting the student evaluation of their clinical placement experience in Ghana. We examined nursing and midwifery students’ evaluation of their clinical placement using the CLES+T.

## Methods

### Study design and setting

This was a cross-sectional study involving undergraduate nursing and midwifery students of the University for Development Studies on clinical placement in health facilities in the Tamale Metropolis.

The Tamale Metropolitan Assembly (normally of a population of ≥250, 000) is one of the 260 Metropolitan, Municipal (normally of a ≥95,000 population) and District (normally of a ≥75,000 population) Assemblies in Ghana. Tamale is the capital town of the metropolis and the Northern region of Ghana. Until 2004, it was a municipality. It is the largest of the 16 metropolitan, municipal and district assemblies in the northern region of Ghana. According to the 2010 population and housing census, the metropolis has a population 223,252 made up of 49.8 and 50.2% males and females respectively. Though the main language of the people is Dagbani, due to its cosmopolitan nature, all the different Ghanaian languages can be heard in the municipality [21].

Apart from the University for Development studies which trains nurses and midwives among other health professionals, the metropolis has two of the oldest nursing training institutions in Ghana; the Tamale Nursing and midwifery training college and the Tamale community health nursing school. One of the three teaching hospitals in Ghana- The Tamale Teaching Hospital, is located in this metropolis. Students are, therefore, placed in this hospital and three other public hospitals (Tamale Central, West and the Seventh Day Adventist hospitals) for the clinical practicum.

### Sampling and clinical placement

Purposive and convenient sampling techniques were used. Purposive, because only students who were toward the end of their studies (third and fourth years’ students) for nursing and midwifery degree were selected to participate in the study. Undergraduate education in Ghana is for 4 years and range from level 100 to level 400. The clinical supervisors sign off the clinical assessment of the students. This assessment constitutes 40% of the mark a student will score in his or her practical exams for the semester. The rest of the 60% is from Objective Structured Clinical Examination (OSCE) conducted by the training institutions (the school). The clinical placement of undergraduate students is divided into intra semester (students are placed for 1 day each week whiles they continue with their lectures and academic activities) and after semester (when students are done with their lectures and examination for the semester and proceed to spend the rest of it clinical placement. This comprise of four to ten weeks block for first and second semesters respectively). This was to ensure that students had enough exposure to clinical placement to enable them to evaluate their experience. However, it was convenient because students in these year levels who were available and willing to participate were selected.

The questionnaire was administered to students by the researchers in the university campus. The questionnaire was self-administered and participants were allowed to take the questionnaires home and return completed copies to the researchers.

### The study questionnaire

We used the English version of the Clinical Learning Environment and Supervision + Nurse Teacher (CLES +T) evaluation scale [22] with prior permission. This psychometric testing scale consists of a total of 34 items within five sub-dimensions. The sub-dimensions are: pedagogical atmosphere on the ward (nine items), leadership style of the ward manager (four items), premises of nursing on the ward (four items), supervisory relationship (eight items), and role of the nurse teacher in clinical practice (nine items). The questions were scored on a five-point Likert scale of 1 to 5. The scores were as follows: 1=fully disagree, 2=disagree to some extent, 3=neither agree nor disagree, 4=agree to some extent and 5=fully agree. We added questions on programme of study and level.

## Statistical analysis

Statistical analyses were done using SPSS version 21. Demographic characteristics of students are presented as frequencies and percentages. Internal consistency was checked for the overall scale and each of the five dimensions using Cronbach’s alpha. There was high internal consistency of the overall CLES +T (Cronbach’s alpha = 0.904). The five dimensions also showed high internal consistency with Cronbach’s alpha values ranging from 0.713 to 0.903 which showed the suitability of the use of this scale (Additional file 1). The method of supervision was categorised into three based on responses to six questions. Unsuccessful supervision was assigned based on a combination of three alternative questions: (i) the student did not have a named supervisor; (ii) a personal supervisor was named, but the relationship with this person did not work; and (iii) the named supervisor changed during the training course. Team supervision was assigned based on a combination of: (i) the supervisor varied according to shift or place and (ii) the supervisor had several students. Successful supervision was where students had a named mentor and the relationship worked in practice [23, 24].

An overall mean score of the questionnaire was calculated for each student by calculating the mean score of all questions. Scores on the five sub-dimensions were also calculated for each student using scores of the questions that make up those dimensions. Higher scores indicate more agreement with the statements.

The association between student demographic characteristics and clinical placement experience and mean scores was determined using t-test or ANOVA as appropriate. We determined associations of the overall mean score on CLES+T and the sub-dimensions using the mean scores (continuous) as dependent variable and demographic characteristics (binary/categorical) and clinical experience (binary/categorical) as independent variables.

## Results

### Background characteristics and clinical experience of students

Majority of the sampled students were undergraduate nursing students (67%) and were in their third year (81%) of study. More than five in ten students received supervision from a nurse (57%) during clinical placement while nurse specialists (4%) provided least supervision. Team supervision (where students are not assigned to specific supervisor but are qualified or registered nurses on duty for the shift do the supervision) (67%) was the most common supervision students received during clinical placement. About three in ten students reported unsuccessful supervision (29%) while only 4% received successful supervision during clinical placement. Moreover, most students did not have one on one contact with their supervisor (46%) and the most frequent private contact with clinical supervisor was once or twice during the course of placement (27%) (Table 1).

**Table 1:** Background characteristics and clinical experience

Characteristic, n (%)	Summary values (***n*** = 225)
**Programme of study**	
BSc. Nursing	150 (66.7)
BSc. Midwifery	75 (33.3)
**Level (year)**	
300 (third)	42 (18.7)
400 (fourth)	183 (81.3)
**Title of clinical supervisor**	
Nurse	129 (57.3)
Nurse specialist	8 (3.6)
Assistant ward manager	16 (7.1)
Sister/ward manager	40 (17.8)
Midwife	32 (14.2)
**Method of supervision**	
Unsuccessful supervision	66 (29.3)
Team supervision	151 (67.1)
Successful supervision	8 (3.6)
**Frequency of private contact with supervisor**	
Not at all	104 (46.2)
Once or twice during the course	61 (27.1)
Less than once a week	10 (4.4)
About once a week	23 (10.2)
More often	27 (12.0)

### Mean scores on the overall scale and sub-dimensions

Students had good perceptions of their clinical placement (mean CLES + T = 3.24). Student perceptions on the sub-divisions of CLES + T varied considerably. The highest score was for the *Leadership style of the ward manager* (3.6) while *Role of the nurse teacher in clinical practice* (3.06) dimension of CLES + T was least scored (Table 2).

**Table 2:** Mean score on the CLES + T and dimensions

Item	Mean (SD)
Total CLES + T	3.24 (0.60)
**Dimensions of CLES + T**	
Pedagogical atmosphere	3.29 (0.72)
Leadership style of the ward manager	3.63 (0.85)
Premise of nursing on the ward	3.19 (0.94)
Supervisory relationship	3.20 (0.96)
Role of the nurse teacher in clinical practice	3.07 (0.84)

### Association between student demographics, clinical experience and mean CLES + T score

The results show that mean CLES + T score was not associated with year of study even though third year students had a little higher scores than fourth year students (3.3 vs 3.2, *p*=0.405). Mean CLES + T score associated weakly with the title of assigned clinical supervisor (*p*=0.063). There was evidence of association between mean score and student programme of study, with nursing students scoring a little higher than midwifery students (*p*=0.002); hence, nursing students perceived their clinical learning environments better than midwifery students. Students who had successful supervision were more likely to have higher scores than those who did not (*p*=0.001). Students who reported increased contact with their private supervisors had higher mean scores (*p=*0.002) (Table 3).

**Table 3:** Association between student demographics, clinical experience and mean CLES + T score

Factor	Mean (SD)	Test statistic
**Programme of study**		
BSc. Nursing	3.15 (0.55)	t = −3.1, *p* =0.002
BSc. Midwifery	3.14 (0.67)	
**Level (year)**		
300 (third)	3.32 (0.75)	t = 0.84, *p* = 0.405
400 (fourth)	3.22 (0.56)	
**Title of clinical supervisor**		
Nurse	3.15 (0.55)	*F =* 2.27, *p* = 0.063
Nurse specialist	3.36 (0.51)	
Assistant ward manager	3.44 (0.53)	
Sister/ward manager	3.42 (0.63)	
Midwife	3.26 (0.75)	
**Method of supervision**		
Unsuccessful supervision	3.07 (0.68)	*F=* 7.38, *p* = 0.001
Team supervision	3.38 (0.54)	
Successful supervision	3.83 (0.43)	
**Frequency of private contact with supervisor**		
Not at all	3.14 (0.61)	*F=* 4.51, *p* = 0.002
Once or twice during the course	3.26 (0.55)	
Less than once a week	2.83 (0.53)	
About once a week	3.46 (0.60)	
More often	3.53 (0.53)	

### Association between student demographics, clinical experience and mean score of the dimensions of CLES + T

We investigated the relationship between mean scores of the five dimensions of CLES + T and student’s demographic factors and clinical placement experience. The data show that method of supervision and frequency of private contact with supervisor were associated with the *Pedagogical atmosphere* dimension of CLES + T. For example, while students who received successful supervision (3.6) had better perceptions of the *Pedagogical atmosphere,* those who had unsuccessful supervision (3.1) and those who received team supervision (3.4) had poor perceptions. Only the method of supervision students received was associated with the *Leadership style of the ward manager* dimension of CLES +T (*p*=0.023). *Premise of nursing on the ward* dimension was associated with the programme of study of students and their frequency of private contact with supervisor (Table 4).

**Table 4:** Association between student demographics, clinical experience and mean score of first three dimensions of CLES + T

Dimension	Mean (SD)	Test statistic
***Pedagogical atmosphere***		
**Method of supervision**		
Unsuccessful supervision	3.06 (0.81)	*F =* 5.07, *p* = 0.007
Team supervision	3.37 (0.66)	
Successful supervision	3.56 (0.76)	
**Frequency of private contact with supervisor**		
Not at all	3.17 (0.76)	*F =* 5.45, *p* < 0.001
Once or twice during the course	3.32 (0.65)	
Less than once a week	2.70 (0.60)	
About once a week	3.64 (0.55)	
More often	3.60 (0.63)	
***Leadership style of the ward manager***		
**Method of supervision**		
Unsuccessful supervision	3.39 (0.84)	*F =* 3.86, *p* = 0.023
Team supervision	3.72 (0.84)	
Successful supervision	3.75 (0.91)	
***Premise of nursing on the ward***		
**Programme of study**		
BSc. Nursing	3.09 (0.93)	t = −2.23, *p* = 0.027
BSc. Midwifery	3.39 (0.96)	
**Frequency of private contact with supervisor**		
Not at all	3.10 (0.93)	*F =* 3.13, *p* = 0.016
Once or twice during the course	3.14 (0.98)	
Less than once a week	2.73 (0.84)	
About once a week	3.35 (0.97)	
More often	3.70 (0.81)	

In addition, *Supervisory relationship* was associated with programme of study (*p*=0.002), frequency of contact with private supervisor (*p*=0.001) and method of supervision received (*p* < 0.001). The last dimension of CLES + T: *Role of nurse teacher in clinical practice was* also associated with programme of study (*p*=0.010) and method of supervision (*p*=0.015) (Table 5).

**Table 5:** Association between student demographics, clinical experience and mean score of last two dimensions of CLES + T

Dimension	Mean (SD)	Test statistic
***Supervisory relationship***		
**Programme of study**		
BSc. Nursing	3.06 (0.93)	t = −3.09, *p =* 0.002
BSc. Midwifery	3.48 (0.96)	
**Frequency of private contact with supervisor**		
Not at all	2.98 (0.93)	*F =* 4.95, *p =* 0.001
Once or twice during the course	3.97 (0.88)	
Less than once a week	3.55 (0.89)	
About once a week	3.49 (0.94)	
More often	3.36 (0.94)	
**Method of supervision**		
Unsuccessful supervision	2.93 (1.10)	*F=* 8.42, *p <* 0.001
Team supervision	3.26 (0.86)	
Successful supervision	4.27 (0.31)	
***Role of the nurse teacher in clinical practice***		
**Programme of study**		
BSc. Nursing	2.97 (0.81)	t = −2.60, *p =* 0.010
BSc. Midwifery	3.27 (0.86)	
**Method of supervision**		
Unsuccessful supervision	3.07 (0.82)	*F =* 4.30, *p* = 0.015
Team supervision	3.02 (0.83)	
Successful supervision	3.90 (0.76)	

## Discussion

In this cross-sectional study among undergraduate nursing and midwifery students in the Tamale metropolis of Ghana, we assessed students’ evaluation of their clinical experience in health facilities using the Clinical Learning Environment and Supervision + Nurse Teacher (CLES +T) evaluation scale. The main findings are that; more students received supervision from nurses during clinical placement and team supervision was the most common supervisory type. Nursing students were more likely to rate their clinical experience better than midwifery students and students who had increased contacts with private supervisors were more likely to rate their experience higher. Clinical experience was also evaluated better by students who received successful supervision compared to those who had other forms of supervision.

The higher likelihood of nurses than other health professionals such as midwives to provide supervision to students on clinical placement in this setting may be explained by the type of skills and competencies students are placed to acquire. This is because many basic skills for both nursing and midwifery as well as advanced skills are taught by nurses as most of those skills are general until students, such as the midwifery students move on to perform specific midwifery skills. The higher rating of clinical placement by nursing students than midwifery students in the present study could mean that midwifery students were expecting that since they are midwives they needed to have been supervised by only midwives; there is also the tendency to view midwifery practice totally separate from nursing practice, which in fact, should not be the case because most basic skills performed in midwifery practice also occur in nursing practice. This is a perception that needs more exploration to inform students’ experience and appreciation of their practice and skills acquisition. It is, therefore, useful for students to be made aware of this ahead of their clinical placement to avoid being unsatisfied with the initial nurse dominant supervision they receive.

We find that frequency of contact with private supervisor was associated with high evaluation of clinical experience by both nursing and midwifery students. These findings are consistent with the results from Cyprus [14], Slovakia [25], northern Italy [26] and Sweden [27] where nursing students evaluated their clinical experience better with private supervision. As team supervision was higher and likely to be poor among students, it is reasonable that contact with private supervisors with a high tendency for tailored training to receive high rating. There is high need for private supervision as revealed in the study and calls for students to make more efforts and make good use of this opportunity whenever available to gain the needed skills. We recommend that clinic or ward staff also endeavour to provide private supervision to increase the quality of clinical experience of students.

Consistent with previous findings [14, 23, 26], successful supervision was associated with higher evaluation of student clinical experience. However, this has not always been the case in all studies [28]. Successful supervision constitutes having a named mentor and a perception of the relationship having worked out. Therefore, it is understandable that students who had successful supervision rated their overall clinical experience better. For improved clinical experience of students, mentors and students should work together to achieve a successful supervision.

To the best of our knowledge, this is the first attempt to report nursing and midwifery students’ evaluation of their clinical placement in Ghana. The findings could be useful to health institutions and providers such as hospitals and clinics involved in the training of nursing and midwifery students to achieve better clinical experience and skills acquisition. However, the results of the study should be interpreted keeping some limitations in mind. First, our sample consisted of more nursing students than midwifery students, so the generalizability of the findings may be less applicable to midwifery students. Second, most supervisors at the ward level were often of a lower educational level than the students which could affect the quality of supervision and supervisory relationship. While this is not a typical limitation of the study, it may have influenced the evaluation of student experience of their clinical placement. Third, the CLES+T questionnaire has not been validated in this setting. However, we do not think this would have affected our results greatly, as the questionnaires were administered in the original English language not translated into a different Ghanaian language. The tests for reliability of the CLES+T using Cronbach’s alpha were also within acceptable ranges indicating its suitability. In spite of these limitations, our results provide important insights into the experiences of nursing and midwifery students during clinical placement in Ghana.

## Conclusion

Team supervision is high in health facilities where students undertake clinical placement in Ghana; nursing students are more likely to rate their clinical experience better than midwifery students and students with increased contacts with private supervisors were more likely to rate their experience better. Frequent contact with private supervisor and successful supervision are associated with better rating of clinical experience among Ghanaian undergraduate nursing and midwifery students.

## Supplementary Information

**Additional file 1.** Test for internal consistency of CLES + T (Cronbach’s alpha).

## Data Availability

The data supporting the conclusions of this article are included within the manuscript. The datasets could be obtained from the corresponding author upon reasonable request.

## Abbreviation

CLES+T: Clinical Learning Environment and Supervision + Nurse Teacher evaluation scale
