# Supplementary material for: Clinical learning environment of nursing and midwifery students in Ghana
Source: BMC Nurs. 2021 Jan 7;20:14. doi: 10.1186/s12912-020-00533-8 (PMC7791834; doi:10.1186/s12912-020-00533-8)
Supplement: Supplementary file 1 — Additional file 1. Test for internal consistency of CLES + T (Cronbach’s alpha). [file 12912_2020_533_MOESM1_ESM.docx]

**Additional file 1: Test for internal consistency of CLES + T (Cronbach’s alpha)**

| **Item** | **Cronbach’s alpha** |
| --- | --- |
| Total CLES + T | 0.904 |
| **Dimensions of CLES + T** |  |
| Pedagogical atmosphere | 0.773 |
| Leadership style of the ward manager | 0.754 |
| Premise of nursing on the ward | 0.713 |
| Supervisory relationship | 0.903 |
| Role of the nurse teacher in clinical practice | 0.848 |
